# Editorial: Not Funny! A [Super] Serious Multidisciplinary Exploration of Humor Creativity

**DOI:** 10.3389/fpsyg.2022.834558

**Published:** 2022-02-03

**Authors:** Maria Elide Vanutelli, Mirella Manfredi, Ori Amir, Claudio Lucchiari

**Affiliations:** ^1^Department of Philosophy Piero Martinetti, University of Milan, Milan, Italy; ^2^Department of Psychology, University of Zurich, Zurich, Switzerland; ^3^Department of Psychology, Pomona College, Claremont, CA, United States

**Keywords:** humor, creativity, fMRI, divergent thinking, comedy, laughter, brain

Creativity and humor are both individual traits and abilities and share many aspects with each other (Luria et al., 2018). Several researchers have pointed to both theoretical and empirical connections between these two constructs (O'Quin and Derks, 1997; Chang et al., 2015; Martin and Ford, 2018) where the elements of incongruity, surprise, originality, and novelty appear to be primarily involved. Consequently, humor has been most often considered a subdomain of creativity [see reviews by Murdock and Ganim (1993), Galloway (1994), and O'Quin and Derks (1997)] and this has been reflected in the use of humor comprehension and production by tests and questionnaires designed to assess creativity (see for example Kaufman Domains of Creativity Scale; Kaufman, 2012; the Creative Achievement Questionnaire; Carson et al., 2005).

However, several aspects concerning the relationship between creativity and humor still remain to be clarified. Specifically, it is not clear what properties characterize and distinguish them and what specialized neural mechanisms underlie them. Therefore, the aim of this Research Topic was to deepen and clarify the processes involved in humor both in relation to creativity, but also as a construct *per se*, across various disciplinary fields.

One of the first scholars discussing the relationship between humor and creativity, Koestler, in his theory of bisociation (Koestler, 1964), claimed that the creation of humor includes a creative act, which consists in fusing together two usually incongruent matrices. This idea is further explored and elaborated in the context of new evidence in the present Research Topic thanks to the contribution of Maraev et al. who proposed the use of four elements in creating humor, which consist of using previous knowledge (1: old) to create something new (2) and original. The authors propose that such a novelty can be also reached by borrowing (3), or importing, unrelated dialog frames into new scenarios or situation. Interestingly, the fourth element concerns the introduction of taboo topics into the jokes (4) both to “spice up” and to lighten the humoristic exchange, which for the authors is characterized as a highly interpersonal act.

The theme of taboo in humor has also been addressed by two other papers in the Research Topic. Bartolo et al. investigated the neural networks underlying black humor with fMRI by comparing participants' reaction to neutral or offensive statements, and disparagement jokes. Results revealed the presence of incongruity in relation to disparagement jokes since the desire to laugh coexists with the awareness of social inappropriateness. This phenomenon seems to involve the activation of two distinct networks: the first one includes the supplementary motor area and the precuneus, and the second involves the anterior cingulate cortex and insula. A key role as a mediator area of this double process is played by the dorsal striatum, which regulates the affective responses aroused by such incongruence. Here, the importance of considering the emotional components of humor is clearly evident.

The paper by Perchtold-Stefan et al., instead, compared the individual humor style of the participants (sarcastic, benevolent, nonsense, and so on) with malevolent creativity, that is the ability to imagine evil and vindictive solutions to unfair problems. The results suggest that people with humor styles motivated by malicious interpersonal goals were also more fluent in malevolent creativity. Motivation, thus, appears to be a possible explanatory factor underlying the two skills.

However, despite the evidence suggesting a relationship between humor and creativity, we believe that humor should not be restricted to just one of its sub-categories. As O'Quin and Derks (1997, p. 245) pointed out: *A creative product is not always funny, and a funny idea is only creative in a very special way, involving originality and a resolution that takes social and human factors into account*. Thus, humor should be considered an independent category, albeit closely related to creativity. In their recent book, Martin and Ford (Martin and Ford, 2018, p. 3) offered the following definition of humor: *Humor is a broad, multifaceted term that represents anything that people say or do that others perceive as funny and tends to make them laugh, as well as the mental processes that go into both creating and perceiving such an amusing stimulus, and also the emotional response of mirth involved in the enjoyment of it*.

Following this direction, the paper by Lin et al. focused on the neural networks underlying humorous movie viewing by applying partial least square analysis to both imaging data and behavioral measures indicating the perceived humorousness of participants. Results shed light on various brain areas involved with humor processing, which include the lateral portion of the prefrontal cortex and the temporo-parietal junction, which has been previously found to be involved in humor perception (Bartolo et al., 2006; Amir et al., 2015), and humor creativity (Amir and Biederman, 2016).

In summary, the papers under this Research Topic highlight both the clear and robust relationship between creativity and humor, and the need to treat the two constructs independently—noting empirical and theoretical distinctions. The papers further highlight the contribution of both cognitive and emotional components in humor processing. Finally, the studies highlight the importance of considering humor as a complex phenomenon involving both personality (e.g., motivational) and interpersonal factors to offer a more ecological and complete understanding of the phenomenon. This means that both the sender and the receiver should be considered a part of the equation. Accordingly, it should be approached and measured by considering psychological, psychophysiological, educational, and social factors.

## Author Contributions

All authors listed have made a substantial, direct, and intellectual contribution to the work and approved it for publication.

## Funding

This research was partially funded by the Department of Philosophy Piero Martinetti of the University of Milan under the Project Departments of Excellence 2018–2022 awarded by the Ministry of Education, University and Research (MIUR).

## Conflict of Interest

The authors declare that the research was conducted in the absence of any commercial or financial relationships that could be construed as a potential conflict of interest.

## Publisher's Note

All claims expressed in this article are solely those of the authors and do not necessarily represent those of their affiliated organizations, or those of the publisher, the editors and the reviewers. Any product that may be evaluated in this article, or claim that may be made by its manufacturer, is not guaranteed or endorsed by the publisher.
